# Solar-Enhanced Advanced Oxidation Processes for Water Treatment: Simultaneous Removal of Pathogens and Chemical Pollutants

**DOI:** 10.3390/ijerph120809542

**Published:** 2015-08-14

**Authors:** Oyuna Tsydenova, Valeriy Batoev, Agniya Batoeva

**Affiliations:** Baikal Institute of Nature Management, Siberian Branch of the Russian Academy of Sciences, Sakhyanova st. 6, Ulan-Ude City 670047, Russia; E-Mails: vbat@binm.bscnet.ru (V.B.); abat@binm.bscnet.ru (A.B.)

**Keywords:** water/wastewater treatment, solar-enhanced AOPs, disinfection, pathogen inactivation, pollutant degradation, simultaneous removal, photo-Fenton, TiO_2_ photocatalysis

## Abstract

The review explores the feasibility of simultaneous removal of pathogens and chemical pollutants by solar-enhanced advanced oxidation processes (AOPs). The AOPs are based on *in-situ* generation of reactive oxygen species (ROS), most notably hydroxyl radicals •OH, that are capable of destroying both pollutant molecules and pathogen cells. The review presents evidence of simultaneous removal of pathogens and chemical pollutants by photocatalytic processes, namely TiO_2_ photocatalysis and photo-Fenton. Complex water matrices with high loads of pathogens and chemical pollutants negatively affect the efficiency of disinfection and pollutant removal. This is due to competition between chemical substances and pathogens for generated ROS. Other possible negative effects include light screening, competitive photon absorption, adsorption on the catalyst surface (thereby inhibiting its photocatalytic activity), *etc.* Besides, some matrix components may serve as nutrients for pathogens, thus hindering the disinfection process. Each type of water/wastewater would require a tailor-made approach and the variables that were shown to influence the processes—catalyst/oxidant concentrations, incident radiation flux, and pH—need to be adjusted in order to achieve the required degree of pollutant and pathogen removal. Overall, the solar-enhanced AOPs hold promise as an environmentally-friendly way to substitute or supplement conventional water/wastewater treatment, particularly in areas without access to centralized drinking water or sewage/wastewater treatment facilities.

## 1. Introduction

According to the latest available estimates, 748 million people worldwide lacked access to potable water in 2012 [1]. Consumption of poor-quality drinking water contaminated with pathogens and chemical pollutants is associated with a number of both short- and long-term adverse health outcomes. For example, diarrhea, often resulting from ingesting pathogens with contaminated drinking water, was the cause of about 1.5 million human deaths in 2012 alone [1]. The major reasons for contaminated drinking water are its inadequate treatment before distribution and contamination of its sources—surface water bodies and shallow groundwater affected by discharges of untreated or inadequately treated sewage/wastewaters. Therefore, provision of efficient treatment methods for both drinking water and sewage/wastewater is a pressing issue, especially in developing countries where a high proportion of population lacks access to improved drinking water. 

The so-called advanced oxidation processes (AOPs) have been drawing attention of researchers and water treatment professionals and were suggested for application in water/wastewater treatment [2,3,4]. The AOPs can be broadly defined as aqueous phase oxidation methods based on *in situ* generation of highly reactive oxygen species (ROS) such as (primarily but not exclusively) hydroxyl radicals [2]. Hydroxyl radical (•OH) is a powerful oxidant species that can oxidize and mineralize almost any chemical compound yielding environmentally benign CO_2_ and inorganic ions [5,6]. The free radicals can also damage microbial cells by attacking cell wall, cytoplasmic membrane and intracellular structures [7].

In some AOPs, such as TiO_2_ photocatalysis and photo-Fenton process, the generation of ROS can be enhanced by light. In recent years, a lot of research is done on the AOPs that can be driven by sunlight [3,4,5,8,9,10,11,12]. The use of renewable and free solar energy in such processes could substantially reduce treatment costs and is more favorable from an environmental perspective [13]. The solar-enhanced methods seem to be particularly suitable for countries located in regions with abundant sunlight, which is the case of many developing countries with drinking water issues. Besides, the ability of AOPs to remove both pathogens and chemical pollutants could further help to improve the economic efficiency of water/wastewater treatment by combining disinfection and pollutant removal—two traditionally separate processes—into one treatment step. 

The application of solar-enhanced AOPs in water/wastewater treatment is a relatively new area of research. There has been not much data published so far on the simultaneous removal of chemical pollutants and pathogens by solar AOPs, although the methods hold promise in the area. The objectives of the review were to (1) gather information on the feasibility and limitations of simultaneous removal of chemical pollutants and pathogens by solar-enhanced AOPs, and (2) draw implications for future research in this direction and developing water/wastewater treatment methods. 

## 2. Literature Search and Selection Criteria

The literature search was restricted to the studies that used either natural sunlight or artificial light sources with emission spectra in UVA-Vis region. Another criterion was the simultaneous presence of chemical pollutants and pathogens in the treated water/wastewater. Although there are a number of publications where either pathogens or chemicals were separately targeted, there are few published reports on the simultaneous removal of chemical pollutants and pathogens using solar-enhanced AOPs. The available reports that met the criteria were few and employed two photocatalytic processes: heterogeneous TiO_2_ photocatalysis and homogeneous photo-Fenton, based on the use of a wide-band gap semiconductor and addition of H_2_O_2_ to dissolved iron salts, respectively. 

## 3. Simultaneous Pathogen Inactivation and Pollutant Degradation

TiO_2_ photocatalysis and photo-Fenton are by far the most studied AOPs that have been shown to be capable of removing chemical pollutants [11,12,14,15,16,17,18] and pathogens, including bacteria, viruses, fungi and protozoa [7,9,10,19,20,21,22]. In TiO_2_ photocatalysis, free hydroxyl •OH radicals are generated upon irradiation of a catalytic semiconductor, such as TiO_2_, with near-UV light of wavelengths < 385 nm [23]:

(1) $$\text{Ti}{\text{O}}_{2}+hv\to \text{ Ti}{\text{O}}_{2}({\text{e}}^{-}+{\text{h}}^{+})$$

(2) $$\text{Ti}{\text{O}}_{2}({\text{h}}^{+})+{\text{H}}_{2}\text{O }\to \text{ Ti}{\text{O}}_{2}+•\text{OH + }{\text{H}}^{+}$$

In photo-Fenton processes, UV-Vis radiation (*λ* ≤ 600 nm) enhances production of hydroxyl radicals via a series of catalytic cycle reactions of iron (Fe^2+^ and Fe^3+^) and H_2_O_2_ [24]:

(3) $${\text{Fe}}^{\text{2+}}+{\text{H}}_{2}{\text{O}}_{2}\to {\text{Fe}}^{\text{3+}}+{\text{OH}}^{-}+\text{•OH}$$

(4) $$\text{Fe}{\text{(OH)}}^{\text{2+}}+hv\to {\text{Fe}}^{\text{2+}}+\text{•OH}$$

Furthermore, several reports have demonstrated that solar-enhanced TiO_2_ photocatalysis and photo-Fenton are capable of simultaneous removal of pathogens and chemical pollutants, at least in laboratory and pilot scale experiments [17,23,25,26,27,28,29,30]. Table 1 and Table 2 present experimental parameters and summarized results from the reviewed reports. Although the simultaneous removal of pathogens and chemical pollutants is an extremely attractive goal, there are certain challenges. Most of the reviewed reports highlight that complex matrices and simultaneous presence of chemical pollutants and pathogens negatively affect the efficiency the photocatalytic processes [23,26,27,29,31]. The phenomenon has been observed in both TiO_2_ photocatalysis and photo-Fenton. Several reasons have been proposed to explain the phenomenon, including the most obvious—competition between chemical pollutants and pathogens for generated ROS. 

**Table 1:** Overview of the studies that employed solar-enhanced TiO_2_ photocatalysis for simultaneous removal of organic compounds and pathogens.

Substrates, Initial Concentration	Experimental Conditions * (Light Source, Reactor Type and Volume)	Results Obtained (Degree of Degradation/Inactivation **, Irradiation Time)	Reference
17α-ethynylestradiol (0.1 mg/L) + *E. coli* (1 × 10^3^ CFU/mL) (in synthetic wastewater)	Solar simulator system,5.8 × 10^−7^ Einstein/L·s Batch-type photoreactor, 300 mL	17α-EE: *ca.* 80%, 90 min *E. coli*: *>* 95%, 90 min The degree of degradation/inactivation was less than in deionized water and when the substrates were treated separately.	[27]
Either Resorcinol/ Hydroquinone (10 mg/L) + Either *E. coli/ S.typhimurium* (10^6^ CFU/mL)	Solar simulator lamp, 1000 W/m^2^ Reactor: Pyrex bottle, 80 mL	Resorcinol: *ca.* 50%, 90 min (in the presence of either *E. coli / S.typhimurium*) Hydroquinone: *ca.* 30%, 120 min (in the presence of either *E. coli / S.typhimurium*) *E. coli*: *ca.* 3 logs, 120 min (in the presence of either Resorcinol/Hydroquinone) *S.typhimurium*: *ca.* 1–2 logs, 120 min (in the presence of either Resorcinol/Hydroquinone) The simultaneous presence of dixydroxybenzenes and bacteria negatively affected both the degradation and inactivation processes.	[26]
Either Resorcinol/ Hydroquinone/ Catechol (2 mmol/L) + *E. coli* (10^7^ CFU/mL)	Solar simulator lamp,1000 W/m^2^ Reactor: Pyrex bottle, 50 mL	Resorcinol/ Hydroquinone/ Catechol: *ca.* 25%, 2 h/*ca.* 12%, 2 h/*ca.* 18%, 2 h *E. coli*: 100%, 40 min (in the presence of either Resorcinol/Hydroquinone/Catechol)	[23]

* In all cases Degussa P25 was used at the concentration of 1 g/L. ** The degrees of degradation/inactivation were devised from figures and are approximate values present here just to provide an idea of the extent of degradation/inactivation.

**Table 2:** Overview of the studies that employed solar-enhanced photo-Fenton for simultaneous removal of organic compounds and pathogens.

Substrates, Initial Concentration	Experimental Conditions (Fenton’s Reagent Concentration, Initial pH, Light Source, Reactor Type And Volume)	Results Obtained (Degree of Degradation/Inactivation *, Irradiation Time)	Reference
Resorcinol (10 mg/L) + *E.faecalis* (10^6^ CFU/mL)	[Fe^2+^] =20 mg/L; [H_2_O_2_] = 50 mg/L pH = 6–7 Natural sunlight, 30 ± 2 W/m^2^ Glass reactor, 250 mL	Resorcinol: 100%, <5 min *E.faecalis*: 100%, 10 min	[29]
Either Resorcinol/ Hydroquinone (10 mg/L) + Either *E. coli/ S.typhimurium, S.sonnei*, (10^6^ CFU/ml)	[Fe^3+^] = 1mg/L; [H_2_O_2_] = 60 mg/L pH = 5.0 (initial)Solar simulator lamp, 1000 W/m^2^ Reactor: Pyrex bottle, 80 mL	Resorcinol: *ca.* 60%–80%, 40 min (in the presence of either *E. coli / S.typhimurium / S.sonnei*) Hydroquinone: *ca.* 55%–90%, 40 min(in the presence of either *E. coli / S.typhimurium / S.sonnei*) *E. coli / S.typhimurium / S.sonnei*: *ca.* 2.5–4 logs, 40 min (in the presence of Resorcinol) / *ca.* 4.5–5.5 logs, 40 min (in the presence of Hydroquinone) The simultaneous presence of dixydroxybenzenes and bacteria negatively affected both the degradation and inactivation processes.	[26]
Either Ofloxacin/ Trimethoprim (100 µg/L) + Enterococci (2.53 × 10^3^ CFU/mL) (in secondary treated WW, 6.29–8.6 mg DOC **/L)	[Fe^2+^] = 5 mg/L; [H_2_O_2_] = 75 mg/L Natural sunlight Reactor: CPC ***, 250 L total volume, 85.4 L irradiated volume, circulation speed 600 L/h	Ofloxacin / Trimethoprim: 100% removal (for both) Enterococci at the start of the experiment: 5.00 × 10^2^ CFU/mL (in the presence of Ofloxacin); 2.67 × 10^2^ CFU/mL (in the presence of Trimethoprim) Enterococci at the end of the experiment: 0 CFU/mL, 180 min (in the presence of either Ofloxacin/Trimethoprim)	[28]

* In some cases, the degrees of degradation/inactivation were devised from figures and are approximate values present here just to provide an idea of the extent of degradation/inactivation. ** Dissolved organic carbon. *** Compound Parabolic Collector.

### 3.1. Effect of the Simultaneous Presence of Chemical Pollutants and Pathogens on Their Degradation/Inactivation

**Figure 1:**
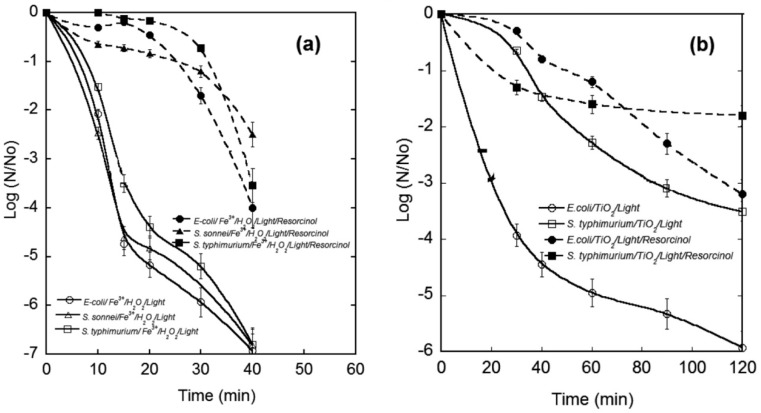
Effect of resorcinol (10 mg/L) on inactivation of bacteria in (**a**) photo-Fenton and (**b**) TiO_2_ photocatalytic processes. Solar simulator light intensity: 1000 W/m, initial bacteria concentration: 10^6^ CFU/mL. Photo-Fenton parameters: Fe^3+^: 1 mg/L, and H_2_O_2_: 60 mg/L, initial pH = 5.0. TiO_2_ photocatalysis: TiO_2_ concentration: 1.0 g/L. Reproduced from [26] with permission of The Royal Society of Chemistry (RSC) on behalf of the Centre National de la Recherche Scientifique (CNRS) and the RSC.

Moncayo-Lasso *et al.* [26] studied the effect of the simultaneous presence of organic compounds (resorcinol and hydroquinone) and bacteria (*Escherichia coli, Salmonella typhimurium* and *Shigella sonnei*) on the degradation of organics and inactivation of bacteria in water by heterogeneous photocatalysis with TiO_2_ and near-neutral photo-Fenton. In both the photocatalytic processes, the degradation of organic compounds and inactivation of bacteria were less efficient when the two substrates were simultaneously present. For example, Figure 1 shows the negative effect of resorcinol on inactivation of bacteria during both the processes. The extent of TiO_2_ photocatalytic degradation of resorcinol and hydroquinone decreased by around 55% and 70%, respectively, when bacteria were simultaneously present. On the other hand, TiO_2_ photocatalytic inactivation of *E. coli* and *S. typhimurium* decreased by *ca.* 3 and *ca.*1–2 logs, respectively, in the presence of the organic compounds. Similar trends were observed when the same substrates were treated by photo-Fenton [26]. In the presence of bacteria, the extent of resorcinol and hydroquinone degradation was only 60%–80% and 55%–90%, respectively, while complete degradation was achieved for both the compounds in the absence of bacteria. At the same time, in the presence of the organic compounds, bacteria inactivation during photo-Fenton was also negatively affected and never reached the 6-log inactivation considered necessary for effective disinfection [5]. The obtained results suggest that in both photocatalytic processes, there is competition between the simultaneously present organic compounds and bacteria for ROS.

Interestingly, Moncayo-Lasso *et al.* [26] have observed that homogeneous photo-Fenton reactions at near-neutral pH were less affected by the simultaneous presence of organics and bacteria than heterogeneous TiO_2_ photocatalytic processes. The authors have attributed the difference to that fact that one process was homogenous, while the other was heterogeneous. Since heterogeneous photocatalytic reactions are taking place largely at the solid–liquid interface, surface-related phenomena, such as adsorption on TiO_2_ and attraction/repulsion between TiO_2_ particles and substrates, play an important role in the efficiency of heterogeneous photocatalytic processes. Such surface-related limitations are not present in homogenous photo-Fenton processes and, therefore, their efficiency is less affected by the simultaneous presence of organics and bacteria. 

In another study employing simulated solar radiation and TiO_2_ as the photocatalyst, the presence of *E. coli* as the second component in the reaction mixture did not obstruct 17α-ethinylestradiol removal [27]. On the other hand, *E. coli* removal was negatively affected by the simultaneously present chemical pollutants. In general, the more complex the water matrix was, the slower *E. coli* removal became. This is due to some non-target species inherently present in the matrix and behaving as scavengers of the photogenerated ROS.

Similar results were obtained by Rincon and Pulgarin when they treated mixtures of *E. coli* and dihydroxybenzenes using TiO_2_ photocatalysis [23]. The presence of dihydroxybenzenes retarded photocatalytic *E. coli* inactivation. However, the effect of *E. coli* presence on the degradation of dihydroxybenzenes, was not examined. During the experimental period, the dihydroxybenzenes were only partially degraded.

Ortega-Gomez *et al.*, have also demonstrated a competition between degradation and disinfection processes during photo-Fenton treatment of *E. coli* and resorcinol [29]. A marked delay in a solar photo-Fenton process of *E. coli* disinfection was observed when resorcinol was present, compared with the same process without resorcinol. The competition was also confirmed by a series of experiments where concentrations of H_2_O_2_/Fe^2+^ or that of resorcinol were gradually increased. *E. coli* inactivation process was disfavored when resorcinol concentrations were increased. Complete inactivation was achieved only with the lowest resorcinol concentration. On the other hand, increasing concentrations of H_2_O_2_/Fe^2+^ lead to improved disinfection. At the highest concentrations of H_2_O_2_/Fe^2+^ (50/20 mg·L^−1^), the disinfection efficiency was independent of the addition of resorcinol. As the photo-Fenton reagent concentrations increased, the amount of generated hydroxyl radicals increased up to the level when the requirements of both processes, *i.e.*, disinfection and degradation, were satisfied. 

Pavelescu *et al.*, have observed the detrimental effect of the simultaneous presence of chemical pollutants and bacteria on the efficiency of TiO_2_ photocatalytic treatment of sewage samples [31]. The different removal efficiency for sewage samples compared with industrial wastewater samples, based on UV-Vis and fluorescence spectroscopy, was attributed to the total coliforms in sewage samples that greatly impact the degree of photocatalytic oxidation.

Polo-Lopez *et al.*, have also observed competition between organic matter and *Fusarium solani* spores for H_2_O_2_, hydroxyl radicals and other oxygen species, during photo-Fenton process [32]. Doubling the concentrations of iron and peroxide hindered inactivation but promoted mineralization, demonstrating competition between spores and the effluent organic matter for hydrogen peroxide, hydroxyl radicals and other ROS.

### 3.2. Effect of Water Matrix on Pathogen Inactivation and Chemical Pollutant Degradation

The chemical composition of water (organic and inorganic) is an important factor that influences not only pathogen inactivation but also degradation of chemical pollutants. Water matrix can be a highly complex mixture of various chemical compounds and its effect on photocatalytic efficiency could be different depending on the compounds present in the water matrix. 

Some organic compounds that are photosensitized by solar radiation have been reported to positively affect efficiency of photocatalytic processes by generating ROS such as ^1^O_2_, O_2_•^−^, HO_2_, H_2_O_2_ or •OH [33]. In [34], the extent of mineralization of dihydroxybenzenes in natural water matrix was higher than in deionized water, suggesting that components of natural water matrix positively affect photo-Fenton process. Spuhler *et al.*, reported resorcinol to facilitate *E. coli* inactivation by photo-Fenton, while inorganic ions present in water matrix generally hindered the process [33]. The authors explained the effect of resorcinol by the formation of Fe^3+^-organo bounds, which undergo photosensitization under solar radiation leading to the generation of ROS. Rodrigues-Chueca *et al.*, reported significantly better inactivation of *E. coli* and *Enterococcus faecalis* in real effluent than in synthetic effluent samples, suggesting that components of the real effluent matrix positively affect the solar photo-Fenton efficiency [24]. Rosado-Lausell *et al.*, reported inactivation of bacteriophage MS2 by ROS and triplet excited state of dissolved organic matter (3DOM *) produced by irradiation of natural and synthetic sensitizers with simulated sunlight [35].

Although some photosensitizing components of water matrix may promote photocatalytic processes, complex water matrices would most probably hinder both the disinfection and pollutant removal. This might be due to the following reasons (extensively discussed in [5] and [23]): 

(1) The organic and inorganic pollutants present in water/wastewater may react with the oxidizing agents, resulting in the non-target consumption of the oxidants. Furthermore, hydroxyl radicals may be scavenged by anions (e.g., bicarbonates, chlorides, sulfates) to form the respective radicals with oxidation potentials lower than that of hydroxyl radicals [5,6,23,24,36,37]. 

(2) The matrix components that absorb light of the same wavelengths as the catalysts may competitively absorb incident photons. This phenomenon is called inner filter effect or screening effect. Although this may lead to some direct photolysis reactions, photolysis reactions normally have a low quantum yield. Hence, photons absorbed by the matrix components instead of the catalyst may be considered lost in terms of efficient photon use [5]. Furthermore, turbidity of real wastewater may impede light transmission through the bulk of the solution thus hindering photocatalysis. 

(3) Certain organic compounds and inorganic ions adsorb onto TiO_2_ surface and inhibit its catalytic activity [23,37]. Among inorganic ions, phosphates and carbonates have been shown to have higher inhibiting capacity of TiO_2_. 

(4) Some organic and inorganic matrix components may serve as nutrients for pathogens, thus helping to maintain their viability and hindering disinfection [23].

The negative effect of water matrix components on photocatalytic processes has been observed in a number of studies [16,23,27,37,38,39,40,41,42]. In a TiO_2_ photocatalytic process, both *E. coli* inactivation and methylene blue oxidation processes were negatively affected by various additives, such as wastewater plant effluent, humic acids, Na_3_PO_4_, NaHCO_3_, and an inorganic–organic mixture (humic acids, Na_3_PO_4_, NaHCO_3_, and NaCl) [39]. Only the addition of NaCl resulted in different responses—*E. coli* inactivation was enhanced, while methylene blue oxidation was impaired. Gogniat *et al.*, reported that chloride anions increase the adsorption of the bacteria on the catalyst compared to other ionic species [43]. Besides, an excess of chloride would promote the inactivation since chlorine-based disinfectant species might be formed, coming from reactions between chloride anions and hydroxyl radicals. Overall, *E. coli* inactivation was found to be more sensitive to the presence of inorganic and organic matter in comparison with the degradation of methylene blue. 

A complex matrix of secondary treated wastewater was shown to have an adverse effect on *E. coli* inactivation, while the removal of 17α-ethinylestradiol was not affected in TiO_2_ photocatalytic treatment [27]. After 90 min of treatment at 500 mg/L of TiO_2_, the disinfection efficiency for real wastewater was three times less than that for synthetic effluent.

Rincon and Pulgarin have extensively experimented with a range of inorganic ions (PO_4_^3−^, HCO_3_^−^, SO_4_^2−^, Cl^−^ and NO_3_^−^) and different water matrices [23,37]. The inorganic ions and complex water matrices have negatively affected TiO_2_ photocatalytic disinfection of *E. coli*. Among the anions, PO_4_^3−^ and HCO^3−^ had the most pronounced negative effect on disinfection. Additions of PO_4_^3−^ have significantly delayed *E. coli* inactivation. The works of the research group have demonstrated that interactions between matrix components, catalyst and bacteria in complex matrices like natural waters are extremely complicated. The water matrix components (inorganic and organic) get involved in hydroxyl radical scavenging, light screening, competitive photon absorption, adsorption on TiO_2_ (thus modifying its photocatalytic activity), reactions with photogenerated holes, *etc.*

There are indications that microbial inactivation processes are more sensitive than chemical oxidation to the composition of the water matrix [23,27,37,39]. Generally, the more complex the water matrix is, the slower the removal of microorganisms becomes. For example, *E. coli* inactivation was affected even at low concentrations (0.2 mmol/L) of SO_4_^2−^ and HCO^3−^, but the same concentrations did not affect resorcinol degradation [39]. This is probably due to that fact that degradation of organic compounds only depends on the photon absorption and hydroxyl radical generation, while microorganisms are also influenced by the osmotic and nutrient effects of the matrix [30,39]. 

Furthermore, inactivation of pathogens is a more complex process than chemical oxidation. Microbiological aspects such as cell repair mechanisms and possible post-experimental re-growth need to be taken into account [39]. Several repair mechanisms have been reported in bacteria, such as photoreactivation, nucleotide excision repair, mutagenic DNA repair, and recombinational DNA repair [23]. Post-experimental re-growth was observed in a number of studies and the lack of residual disinfecting effect of solar photocatalytic treatment is often considered the drawback for wider application of the methods [44]. It is therefore important to ensure complete disinfection as re-growth was observed when phototreatment was stopped in the middle [45]. Giannakis *et al.*, also observed post-irradiation survival/re-growth in different water matrices with kinetic profiles largely determined by the applied light dose [44]. 

## 4. Overcoming the Detrimental Effects of the Simultaneous Presence of Chemical Pollutants and Pathogens 

The above-discussed detrimental effects of the simultaneous presence of chemical pollutants and pathogens, as well as the interference of matrix components need to be overcome in order to achieve a required degree of disinfection and pollutant removal. The complexity typical for real wastewater matrices and the associated obstacles to application of solar AOPs need to be addressed while designing treatment methods. The task can be tackled by optimizing the experimental/operational variables that were shown to influence the processes. These include catalyst/oxidant concentrations, incident radiation flux, and pH. 

### 4.1. Catalyst Concentration

The photocatalytic processes of pathogen inactivation and oxidation of chemical pollutants are activated and governed by the same physicochemical phenomena. Both photo-Fenton and TiO_2_ photocatalytic processes are based on *in-situ* generation of hydroxyl radicals upon solar activation of the catalysts. In both the processes, the catalyst concentration and incident radiation flux influence the common stages of radiation absorption and generation of ROS. Generally, bacteria and chemical pollutants have been shown to respond similarly to the changes in the operational variables that determine the amount of produced hydroxyl radicals [39,46]. 

Both pathogen inactivation and pollutant degradation processes have been shown to intensify with increasing catalyst concentration until a certain catalyst concentration, beyond which there is no further progress in inactivation/degradation. This catalyst concentration is the concentration needed to absorb all photons available under certain experimental conditions. This optimum catalyst concentration depends on the incident radiation flux and reactor geometry, but independent of the target pollutant unless it competes for photons [5]. It is important to find the optimum catalyst concentration in order to avoid excess catalyst, which may result in high turbidity and impede light penetration into the bulk of the treated solution. 

**Figure 2:**
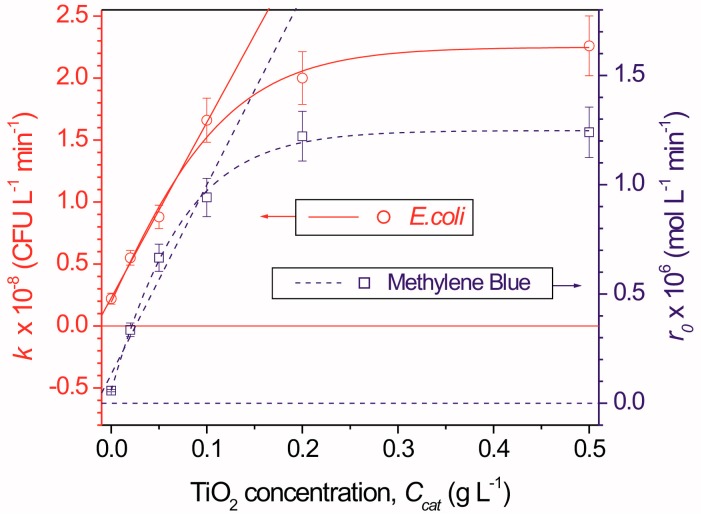
Kinetic constant for *E. coli* inactivation and initial reaction rate of methylene blue oxidation as a function of TiO_2_ concentration. Reproduced from [39] with permission from Elsevier.

Marugán *et al.* [39] and Chen *et al.* [46] have observed a good correlation between TiO_2_ photocatalytic processes of pollutant oxidation (methylene blue and formaldehyde, respectively) and *E. coli* inactivation, when analyzing the effect of catalyst concentration and incident radiation flux. The reason for these similarities seems to be due to the common steps of photon absorption and subsequent generation of •OH radicals, which are independent of the type of pollutant. Figure 2 shows kinetic constant for *E. coli* inactivation and initial reaction rate of methylene blue oxidation as a function of TiO_2_ concentration. It is clearly seen that for both processes the optimum TiO_2_ concentration is around 0.1 g/L.

Furthermore, a great deal of research is being done that involves modifying structure and composition of TiO_2_ in order to enhance its catalytic activity. The research efforts focus on improving visible light absorption and charge separation properties of TiO_2_. Interesting and promising developments in the field that could significantly improve performance characteristics of TiO_2_ include non-metal doping and nanostructuring of the catalyst [47,48,49]. However, this topic is beyond the scope of this review. 

In photo-Fenton, an increase in iron concentration also leads to an increase in inactivation and/or degradation efficiency [29,40,50,51,52,53]. For example, when Fe^2+^ concentration was increased from 1 to 5 mg/L, conversion rate of 17α-ethinilestradiol increased proportionally [52]. In another study, an increase in iron concentration from 2 to 20 mg/L reduced the time required for 80% degradation of initial dissolved organic carbon (DOC) by a factor of 6 [51].

Similarly to TiO_2_ photocatalysis, for a given set of photo-Fenton process parameters there is an optimum iron concentration, beyond which an increase in iron concentration does not lead to a proportional increase in photocatalytic efficiency. For example, increasing Fe^2+^ concentrations in photo-Fenton processes with three oxidants—persulfate, peroxymonosulfate, and hydrogen peroxide—did not result in a proportional increase in degradation rate constant of atrazine [40]. Again, similarly to TiO_2_, this concentration also seems to be dependent on the experimental conditions determining irradiation dose, such as optical path length of the reactor [5]. 

Carra *et al.*, (2014) have experimented with different iron dosage regimes—sequential and continuous iron additions—during photo-Fenton applied to a mixture of pesticides [53]. The dosage regimes allowed pollutant removal at natural pH. Furthermore, the continuous dosage that allows iron to be better distributed resulted in a better removal of the pesticides. The least reactive pesticide among treated was removed in less than 15 min when a continuous exponentially decreasing iron dosage was applied. 

### 4.2. Irradiance

**Figure 3:**
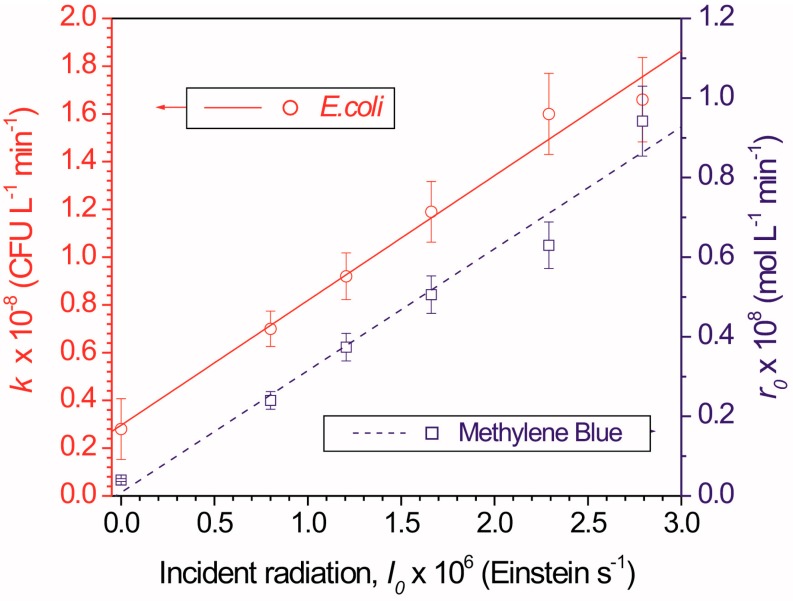
Kinetic constant for *E. coli* inactivation and initial reaction rate of methylene blue oxidation as a function of incident radiation. Reproduced from [39] with permission from Elsevier.

It is well established that increasing light intensity accelerates photocatalytic processes. For example, Rincon and Pulgarin observed enhanced TiO_2_ photocatalytic inactivation of *E. coli* when light intensity increased from 400 to 1000 W/m^2^ [23]. An apparent correlation between the two photocatalytic processes of decomposing formaldehyde and inactivating *E. coli* with respect to light intensity was observed by Chen *et al.* [46]. Figure 3 demonstrates a similar result from another study [39] for *E. coli* inactivation and methylene blue oxidation. Within the examined ranges of irradiation flux, the photocatalytic efficiency of the processes was directly proportional to the radiation dose.

Carra *et al.*, observed UV-light saturation effect, *i.e.*, photo-Fenton the extent of degradation of three target pollutants has increased when the light intensity rose from 5 to 15 W/m^2^, but further increase of intensity beyond 15 W/m^2^ has not resulted in enhanced degradation [54]. Authors concluded that the photon absorption is limited by the reactor configuration (light path length of 5 cm) and low initial concentrations of the target pollutants. 

In solar-driven processes, the irradiance cannot be controlled and irradiation time may need to be adjusted to ensure sufficient photon absorption. One parameter that allows taking into account variable solar irradiation and comparing different solar photocatalytic experiments is “solar UV dose”. The solar UV dose is the solar energy (J/m^2^) received during a photocatalytic treatment, which is defined by solar intensity, expressed as irradiance (W/m^2^), and irradiation time (s) [45,55]. However, there were somewhat contradictory results of applying solar UV dose to characterize performance of TiO_2_ photocatalytic disinfection [45,55]. Rincon and Pulgarin state that UV solar dose is not an appropriate parameter to predict/standardize solar disinfection and seasonal and intraday variations in irradiance significantly affect photocatalytic processes [45]. On the other hand, Sichel *et al.*, have found that there is a certain solar UV dose necessary to reach a certain disinfection level, which depends on the microorganism and the reactor configuration [55]. They have also suggested that solar photocatalytic disinfection does not depend proportionally on solar irradiance as long as enough photons have been received. 

### 4.3. Oxidant Concentration

The efficiency of TiO_2_ photocatalytic processes can be improved by the addition of chemical oxidants, such as hydrogen peroxide and peroxodisulfate [50,56]. Peroxodisulfate seems to be a more efficient oxidant that H_2_O_2_. Higher removal of DOC and chemical oxygen demand (COD) was achieved with peroxodisulfate in comparison to H_2_O_2_ [50,56]. Pathogen inactivation was also reported to be promoted by increasing concentrations of H_2_O_2_ [29]. 

Hydrogen peroxide and persulfate/peroxymonosulfate are the oxidants in the classic Fenton and Fenton-like oxidative systems, respectively. Increasing concentrations of the oxidants were reported to be associated with improved efficiency of the photo-Fenton processes [40,52,57,58]. Increasing H_2_O_2_ concentration were shown to positively affect 17α-ethinilestradiol degradation during a photo-Fenton process [52]. 

For both the photocatalytic processes discussed here there are optimum oxidant concentrations that depend on the substrate and could be empirically determined. A low oxidant concentration would result in a low reaction rate, while a too high concentration would lead to radical scavenging/recombination. Khan *et al.*, have examined the effect of increasing initial concentrations of hydrogen peroxide, persulfate or peroxymonosulfate on degradation rate constant of atrazine in photo-Fenton processes [40]. The degradation rate constants for atrazine increased with increasing oxidant concentrations, although for all oxidants the slopes of the graphs reflecting the relationships have declined at concentrations higher than 40 µM. 

### 4.4. pH

Efficiency of both TiO_2_ photocatalytic and photo-Fenton processes is greatly affected by pH of the treated solution. In the former case, pH defines the surface charge of TiO_2_ particles and thereby affects the degree of attraction/repulsion between the catalyst particles and substrates. Since heterogeneous photocatalytic reactions are taking place largely at the solid-liquid interface, the surface-related phenomena, such as adsorption onto TiO_2_ and attraction/repulsion between TiO_2_ particles and substrates, play an important role in the efficiency of the photocatalytic processes. As hydroxyl radicals are formed on the illuminated semiconductor surface, adsorption onto or attraction of substrates to TiO_2_ particles would favor oxidation/disinfection of the substrates. Using transmission electron microscopy, Nadtochenko *et al.*, have shown that aggregated TiO_2_ particles interact with bacteria cells during the photocatalytic process leading to bacterial lysis [59]. 

Rincon and Pulgarin have examined the effect of pH on surface-related phenomena in TiO_2_ photocatalytic systems [37]. Bacterial cell surfaces possess net negative electrostatic charge due to ionized phosphoryl and carboxylate substituents on outer cell envelope macromolecules, which are exposed to the extracellular environment [60]. If pH of the solution is lower than the point of zero charge of TiO_2_, which is pH 6.5, there are more TiOH_2_^+^ species on TiO_2_ surface. In such a case, the positively charged TiO_2_ particles and negatively charged bacteria would be attracted. If pH of the solution is higher than the point of zero charge of TiO_2,_ there are more TiO^−^ species on the TiO_2_ surface making it negatively charged. Consequently, there would be repulsion between the negatively charged bacteria and TiO_2_ resulting in a lower disinfection rate.

In Fenton processes, pH has a major effect on process efficiency. The highest photo-Fenton efficiency is observed at pH 2.8 [61]. However, using such a low pH in real wastewater treatment processes would present a significant setback, *i.e.* increased operational costs associated with acidification of wastewater prior to treatment and neutralization afterwards. Therefore, researchers have been searching for the ways of avoiding acidification [16,17,24,25,32]. The results have been encouraging. For example, Klamerth *et al.*, have shown that emerging pollutants at low concentrations (µg/L range) can be successfully degraded to negligible concentrations with solar photo-Fenton at low iron concentrations (5 mg/L) and low initial H_2_O_2_ (50 mg/L) concentrations without adjusting pH [16]. Moncayo-Lasso *et al.*, (2009) applied a photo-Fenton process at “natural” pH (6.5) to river water and observed 55% DOC removal (from the initial concentration of 5.3 mg/L) and complete inactivation of *E. coli* without re-growth 24 hours following the treatment [25]. Rodrigues-Chueca *et al.*, showed that, at near neutral pH, low concentrations of dissolved iron (0.2–0.3 mg/L) can produce enough oxidative damage to achieve complete inactivation of bacteria (*E. coli* and *E.faecalis*) [24]. The authors have also found that precipitated iron blocks some of the light entering the reactor and does not provide extra hydroxyl radicals via photo-Fenton reactions.

Overall, the parameters examined in the section—catalyst/oxidant concentrations, incident radiation flux, and pH—would greatly affect the final treatment efficiency and need to be optimized for a particular type of water/wastewater taking into account its qualitative and quantitative composition. Furthermore, the characteristics of the water/wastewater to be treated might require solar-enhanced AOPs to be used in combination with other treatment methods. Solar-enhanced AOPs alone can possibly be used for treatment of relatively unpolluted ground or surface water to be further used for drinking purpose. Heavily polluted sewage and industrial wastewaters would require the AOPs to be used in combination with other treatment methods, *i.e.*, the integration of solar AOPs as part of a treatment train. Solar AOPs have been suggested as a treatment step either preceding or following biological treatment, during which recalcitrant pollutants and pathogens are removed [12,17]. 

## 5. Conclusions

The available scientific data suggest that simultaneous removal of pathogens and chemical pollutants can be achieved using solar-enhanced AOPs—TiO_2_ photocatalysis and photo-Fenton. The combination of solar radiation with AOPs can constitute an environmentally-friendly alternative or a supplement to the conventional treatment methods. The solar-enhanced AOPs have an advantage of using a free and renewable energy source—natural sunlight. The two best studied methods—TiO_2_ photocatalysis and photo-Fenton—have been shown to be capable of simultaneously inactivating microorganisms present at initial concentrations of up to 10^6^ CFU/mL and degrading organic pollutants at concentrations of up to mg/L.

However, the simultaneous presence of chemical pollutants, pathogens and water matrix components presents certain challenges. Chemical pollutants and pathogens compete for generated ROS, thereby negatively affecting their degradation/inactivation efficiency. Besides, adverse interference may arise from the presence of inorganic ions and organic matter in water matrix. Although some photosensitizing components of water matrix may promote photocatalytic processes, complex water matrices (such as industrial wastewater or sewage) tend to hinder both the pathogen inactivation and pollutant removal through hydroxyl radical scavenging, light screening, competitive photon absorption, adsorption onto the catalyst (thereby inhibiting its photocatalytic activity), reactions with photogenerated holes, *etc.* Besides, some matrix components may serve as nutrients for pathogens, thus hindering the disinfection process. 

The complexity typical for real wastewater matrices and the associated obstacles to application of solar AOPs need to be addressed while designing treatment methods. The detrimental effects of the simultaneous presence of chemical pollutants and pathogens, as well as the interference of matrix components need to be overcome in order to achieve the required degree of disinfection and pollutant removal. The task can be tackled by optimizing the variables that were shown to influence the processes—catalyst/oxidant concentrations, incident radiation flux, and pH. The matrix composition should be characterized and addressed with the best-suited set of the variables, optimized for the particular type of wastewater/water. 

More scientific hard data need to be generated in order to facilitate scaling up and commercial application of the solar-enhanced AOPs. The presently available reports on possible applications of the processes for simultaneous removal of pathogens and chemical pollutants are scarce. Further research on the application of solar AOPs for water/wastewater treatment needs to take into account the qualitative and quantitative composition of real water/wastewater. The range and concentrations of model compounds, microorganisms and matrix components in laboratory experiments need to be close to the concentrations usually encountered in real water/wastewater. This would help to better simulate the real water/wastewater and examine synergistic and antagonistic effects between chemical pollutants, microorganisms and matrix components. 
